# Case study research for better evaluations of complex interventions: rationale and challenges

**DOI:** 10.1186/s12916-020-01777-6

**Published:** 2020-11-10

**Authors:** Sara Paparini, Judith Green, Chrysanthi Papoutsi, Jamie Murdoch, Mark Petticrew, Trish Greenhalgh, Benjamin Hanckel, Sara Shaw

**Affiliations:** 1 Nuffield Department of Primary Care Health Sciences, University of Oxford, Oxford, UK; 2 Wellcome Centre for Cultures & Environments of Health, University of Exeter, Exeter, UK; 3 School of Health Sciences, University of East Anglia, Norwich, UK; 4 Public Health, Environments and Society, London School of Hygiene & Tropical Medicin, London, UK; 5 Institute for Culture and Society, Western Sydney University, Penrith, Australia

**Keywords:** Complexity, Context, Evaluation, Qualitative, Case studies, Mixed-method, Public health, Health services research, Interventions

## Abstract

**Background:**

The need for better methods for evaluation in health research has been widely recognised. The ‘complexity turn’ has drawn attention to the limitations of relying on causal inference from randomised controlled trials alone for understanding whether, and under which conditions, interventions in complex systems improve health services or the public health, and what mechanisms might link interventions and outcomes. We argue that case study research—currently denigrated as poor evidence—is an under-utilised resource for not only providing evidence about context and transferability, but also for helping strengthen causal inferences when pathways between intervention and effects are likely to be non-linear.

**Main body:**

Case study research, as an overall approach, is based on in-depth explorations of complex phenomena in their natural, or real-life, settings. Empirical case studies typically enable dynamic understanding of complex challenges and provide evidence about causal mechanisms and the necessary and sufficient conditions (contexts) for intervention implementation and effects. This is essential evidence not just for researchers concerned about internal and external validity, but also research users in policy and practice who need to know what the likely effects of complex programmes or interventions will be in their settings. The health sciences have much to learn from scholarship on case study methodology in the social sciences. However, there are multiple challenges in fully exploiting the potential learning from case study research. First are misconceptions that case study research can only provide exploratory or descriptive evidence. Second, there is little consensus about what a case study is, and considerable diversity in how empirical case studies are conducted and reported. Finally, as case study researchers typically (and appropriately) focus on thick description (that captures contextual detail), it can be challenging to identify the key messages related to intervention evaluation from case study reports.

**Conclusion:**

Whilst the diversity of published case studies in health services and public health research is rich and productive, we recommend further clarity and specific methodological guidance for those reporting case study research for evaluation audiences.

## Background

The need for methodological development to address the most urgent challenges in health research has been well-documented. Many of the most pressing questions for public health research, where the focus is on system-level determinants [1, 2], and for health services research, where provisions typically vary across sites and are provided through interlocking networks of services [3], require methodological approaches that can attend to complexity. The need for methodological advance has arisen, in part, as a result of the diminishing returns from randomised controlled trials (RCTs) where they have been used to answer questions about the effects of interventions in complex systems [4–6]. In conditions of complexity, there is limited value in maintaining the current orientation to experimental trial designs in the health sciences as providing ‘gold standard’ evidence of effect.

There are increasing calls for methodological pluralism [7, 8], with the recognition that complex intervention and context are not easily or usefully separated (as is often the situation when using trial design), and that system interruptions may have effects that are not reducible to linear causal pathways between intervention and outcome. These calls are reflected in a shifting and contested discourse of trial design, seen with the emergence of realist [9], adaptive and hybrid (types 1, 2 and 3) [10, 11] trials that blend studies of effectiveness with a close consideration of the contexts of implementation. Similarly, process evaluation has now become a core component of complex healthcare intervention trials, reflected in MRC guidance on how to explore implementation, causal mechanisms and context [12].

Evidence about the context of an intervention is crucial for questions of external validity. As Woolcock [4] notes, even if RCT designs are accepted as robust for maximising internal validity, questions of transferability (how well the intervention works in different contexts) and generalisability (how well the intervention can be scaled up) remain unanswered [5, 13]. For research evidence to have impact on policy and systems organisation, and thus to improve population and patient health, there is an urgent need for better methods for strengthening external validity, including a better understanding of the relationship between intervention and context [14].

Policymakers, healthcare commissioners and other research users require credible evidence of relevance to their settings and populations [15], to perform what Rosengarten and Savransky [16] call ‘careful abstraction’ to the locales that matter for them. They also require robust evidence for understanding complex causal pathways. Case study research, currently under-utilised in public health and health services evaluation, can offer considerable potential for strengthening faith in both external and internal validity. For example, in an empirical case study of how the policy of free bus travel had specific health effects in London, UK, a quasi-experimental evaluation (led by JG) identified how important aspects of context (a good public transport system) and intervention (that it was universal) were necessary conditions for the observed effects, thus providing useful, actionable evidence for decision-makers in other contexts [17].

The overall approach of case study research is based on the in-depth exploration of complex phenomena in their natural, or ‘real-life’, settings. Empirical case studies typically enable dynamic understanding of complex challenges rather than restricting the focus on narrow problem delineations and simple fixes. Case study research is a diverse and somewhat contested field, with multiple definitions and perspectives grounded in different ways of viewing the world, and involving different combinations of methods. In this paper, we raise awareness of such plurality and highlight the contribution that case study research can make to the evaluation of complex system-level interventions. We review some of the challenges in exploiting the current evidence base from empirical case studies and conclude by recommending that further guidance and minimum reporting criteria for evaluation using case studies, appropriate for audiences in the health sciences, can enhance the take-up of evidence from case study research.

## Main body

### Case study research offers evidence about context, causal inference in complex systems and implementation

Well-conducted and described empirical case studies provide evidence on context, complexity and mechanisms for understanding how, where and why interventions have their observed effects. Recognition of the importance of context for understanding the relationships between interventions and outcomes is hardly new. In 1943, Canguilhem berated an over-reliance on experimental designs for determining universal physiological laws: ‘As if one could determine a phenomenon’s essence apart from its conditions! As if conditions were a mask or frame which changed neither the face nor the picture!’ ([18] p126). More recently, a concern with context has been expressed in health systems and public health research as part of what has been called the ‘complexity turn’ [1]: a recognition that many of the most enduring challenges for developing an evidence base require a consideration of system-level effects [1] and the conceptualisation of interventions as interruptions in systems [19].

The case study approach is widely recognised as offering an invaluable resource for understanding the dynamic and evolving influence of context on complex, system-level interventions [20–23]. Empirically, case studies can directly inform assessments of where, when, how and for whom interventions might be successfully implemented, by helping to specify the necessary and sufficient conditions under which interventions might have effects and to consolidate learning on how interdependencies, emergence and unpredictability can be managed to achieve and sustain desired effects. Case study research has the potential to address four objectives for improving research and reporting of context recently set out by guidance on taking account of context in population health research [24], that is to (1) improve the appropriateness of intervention development for specific contexts, (2) improve understanding of ‘how’ interventions work, (3) better understand how and why impacts vary across contexts and (4) ensure reports of intervention studies are most useful for decision-makers and researchers.

However, evaluations of complex healthcare interventions have arguably not exploited the full potential of case study research and can learn much from other disciplines. For evaluative research, exploratory case studies have had a traditional role of providing data on ‘process’, or initial ‘hypothesis-generating’ scoping, but might also have an increasing salience for explanatory aims. Across the social and political sciences, different *kinds* of case studies are undertaken to meet diverse *aims* (description, exploration or explanation) and across different *scales* (from small N qualitative studies that aim to elucidate processes, or provide thick description, to more systematic techniques designed for medium-to-large N cases).

Case studies with explanatory aims vary in terms of their positioning within mixed-methods projects, with designs including (but not restricted to) (1) single N of 1 studies of interventions in specific contexts, where the overall design is a case study that may incorporate one or more (randomised or not) comparisons over time and between variables within the case; (2) a series of cases conducted or synthesised to provide explanation from variations between cases; and (3) case studies of particular settings within RCT or quasi-experimental designs to explore variation in effects or implementation.

Detailed qualitative research (typically done as ‘case studies’ within process evaluations) provides evidence for the plausibility of mechanisms [25], offering theoretical generalisations for how interventions may function under different conditions. Although RCT designs reduce many threats to internal validity, the mechanisms of effect remain opaque, particularly when the causal pathways between ‘intervention’ and ‘effect’ are long and potentially non-linear: case study research has a more fundamental role here, in providing detailed observational evidence for causal claims [26] as well as producing a rich, nuanced picture of tensions and multiple perspectives [8].

Longitudinal or cross-case analysis may be best suited for evidence generation in system-level evaluative research. Turner [27], for instance, reflecting on the complex processes in major system change, has argued for the need for methods that integrate learning across cases, to develop theoretical knowledge that would enable inferences beyond the single case, and to develop generalisable theory about organisational and structural change in health systems. Qualitative Comparative Analysis (QCA) [28] is one such formal method for deriving causal claims, using set theory mathematics to integrate data from empirical case studies to answer questions about the configurations of causal pathways linking conditions to outcomes [29, 30].

Nonetheless, the single N case study, too, provides opportunities for theoretical development [31], and theoretical generalisation or analytical refinement [32]. How ‘the case’ and ‘context’ are conceptualised is crucial here. Findings from the single case may seem to be confined to its intrinsic particularities in a specific and distinct context [33]. However, if such context is viewed as exemplifying wider social and political forces, the single case can be ‘telling’, rather than ‘typical’, and offer insight into a wider issue [34]. Internal comparisons within the case can offer rich possibilities for logical inferences about causation [17]. Further, case studies of any size can be used for theory testing through refutation [22]. The potential lies, then, in utilising the strengths and plurality of case study to support theory-driven research within different methodological paradigms.

Evaluation research in health has much to learn from a range of social sciences where case study methodology has been used to develop various kinds of causal inference. For instance, Gerring [35] expands on the within-case variations utilised to make causal claims. For Gerring [35], case studies come into their own with regard to invariant or strong causal claims (such as X is a necessary and/or sufficient condition for Y) rather than for probabilistic causal claims. For the latter (where experimental methods might have an advantage in estimating effect sizes), case studies offer evidence on mechanisms: from observations of X affecting Y, from process tracing or from pattern matching. Case studies also support the study of emergent causation, that is, the multiple interacting properties that account for particular and unexpected outcomes in complex systems, such as in healthcare [8].

Finally, efficacy (or beliefs about efficacy) is not the only contributor to intervention uptake, with a range of organisational and policy contingencies affecting whether an intervention is likely to be rolled out in practice. Case study research is, therefore, invaluable for learning about contextual contingencies and identifying the conditions necessary for interventions to become normalised (i.e. implemented routinely) in practice [36].

### The challenges in exploiting evidence from case study research

At present, there are significant challenges in exploiting the benefits of case study research in evaluative health research, which relate to status, definition and reporting. Case study research has been marginalised at the bottom of an evidence hierarchy, seen to offer little by way of explanatory power, if nonetheless useful for adding descriptive data on process or providing useful illustrations for policymakers [37]. This is an opportune moment to revisit this low status. As health researchers are increasingly charged with evaluating ‘natural experiments’—the use of face masks in the response to the COVID-19 pandemic being a recent example [38]—rather than interventions that take place in settings that can be controlled, research approaches using methods to strengthen causal inference that does not require randomisation become more relevant.

A second challenge for improving the use of case study evidence in evaluative health research is that, as we have seen, what is meant by ‘case study’ varies widely, not only across but also within disciplines. There is indeed little consensus amongst methodologists as to how to define ‘a case study’. Definitions focus, variously, on small sample size or lack of control over the intervention (e.g. [39] p194), on in-depth study and context [40, 41], on the logic of inference used [35] or on distinct research strategies which incorporate a number of methods to address questions of ‘how’ and ‘why’ [42]. Moreover, definitions developed for specific disciplines do not capture the range of ways in which case study research is carried out across disciplines. Multiple definitions of case study reflect the richness and diversity of the approach. However, evidence suggests that a lack of consensus across methodologists results in some of the limitations of published reports of empirical case studies [43, 44]. Hyett and colleagues [43], for instance, reviewing reports in qualitative journals, found little match between methodological definitions of case study research and how authors used the term.

This raises the third challenge we identify that case study reports are typically not written in ways that are accessible or useful for the evaluation research community and policymakers. Case studies may not appear in journals widely read by those in the health sciences, either because space constraints preclude the reporting of rich, thick descriptions, or because of the reported lack of willingness of some biomedical journals to publish research that uses qualitative methods [45], signalling the persistence of the aforementioned evidence hierarchy. Where they do, however, the term ‘case study’ is used to indicate, interchangeably, a qualitative study, an N of 1 sample, or a multi-method, in-depth analysis of one example from a population of phenomena. Definitions of what constitutes the ‘case’ are frequently lacking and appear to be used as a synonym for the settings in which the research is conducted. Despite offering insights for evaluation, the primary aims may not have been evaluative, so the implications may not be explicitly drawn out. Indeed, some case study reports might properly be aiming for thick description without necessarily seeking to inform about context or causality.

### Acknowledging plurality and developing guidance

We recognise that definitional and methodological plurality is not only inevitable, but also a necessary and creative reflection of the very different epistemological and disciplinary origins of health researchers, and the aims they have in doing and reporting case study research. Indeed, to provide some clarity, Thomas [46] has suggested a typology of subject/purpose/approach/process for classifying aims (e.g. evaluative or exploratory), sample rationale and selection and methods for data generation of case studies. We also recognise that the diversity of methods used in case study research, and the necessary focus on narrative reporting, does not lend itself to straightforward development of formal quality or reporting criteria.

Existing checklists for reporting case study research from the social sciences—for example Lincoln and Guba’s [47] and Stake’s [33]—are primarily orientated to the quality of narrative produced, and the extent to which they encapsulate thick description, rather than the more pragmatic issues of implications for intervention effects. Those designed for clinical settings, such as the CARE (CAse REports) guidelines, provide specific reporting guidelines for medical case reports about single, or small groups of patients [48], not for case study research.

The Design of Case Study Research in Health Care (DESCARTE) model [44] suggests a series of questions to be asked of a case study researcher (including clarity about the philosophy underpinning their research), study design (with a focus on case definition) and analysis (to improve process). The model resembles toolkits for enhancing the quality and robustness of qualitative and mixed-methods research reporting, and it is usefully open-ended and non-prescriptive. However, even if it does include some reflections on context, the model does not fully address aspects of context, logic and causal inference that are perhaps most relevant for evaluative research in health.

Hence, for evaluative research where the aim is to report empirical findings in ways that are intended to be pragmatically useful for health policy and practice, this may be an opportune time to consider how to best navigate plurality around what is (minimally) important to report when publishing empirical case studies, especially with regards to the complex relationships between context and interventions, information that case study research is well placed to provide.

## Conclusion

The conventional scientific quest for certainty, predictability and linear causality (maximised in RCT designs) has to be augmented by the study of uncertainty, unpredictability and emergent causality [8] in complex systems. This will require methodological pluralism, and openness to broadening the evidence base to better understand both causality in and the transferability of system change intervention [14, 20, 23, 25]. Case study research evidence is essential, yet is currently under exploited in the health sciences. If evaluative health research is to move beyond the current impasse on methods for understanding interventions as interruptions in complex systems, we need to consider in more detail how researchers can conduct and report empirical case studies which do aim to elucidate the contextual factors which interact with interventions to produce particular effects. To this end, supported by the UK’s Medical Research Council, we are embracing the challenge to develop guidance for case study researchers studying complex interventions. Following a meta-narrative review of the literature, we are planning a Delphi study to inform guidance that will, at minimum, cover the value of case study research for evaluating the interrelationship between context and complex system-level interventions; for situating and defining ‘the case’, and generalising from case studies; as well as provide specific guidance on conducting, analysing and reporting case study research. Our hope is that such guidance can support researchers evaluating interventions in complex systems to better exploit the diversity and richness of case study research.

## Data Availability

Not applicable (article based on existing available academic publications)

## Abbreviations

QCA: Qualitative comparative analysis
QED: Quasi-experimental design
RCT: Randomised controlled trial
